# Effects of Glucocorticoids on Postoperative Neurocognitive Disorders in Adult Patients: A Systematic Review and Meta-Analysis

**DOI:** 10.3389/fnagi.2022.939848

**Published:** 2022-06-30

**Authors:** Xiaoyu Xie, Rui Gao, Hai Chen, Xueying Zhang, Xingwei Cai, Changteng Zhang, Changliang Liu, Tao Zhu, Chan Chen

**Affiliations:** ^1^Department of Anesthesiology and National Clinical Research Center for Geriatrics, West China Hospital, Sichuan University and the Research Units of West China (2018RU012), Chinese Academy of Medical Sciences, Chengdu, China; ^2^Laboratory of Anesthesia and Critical Care Medicine, National-Local Joint Engineering Research Centre of Translational Medicine of Anesthesiology, West China Hospital, Sichuan University, Chengdu, China; ^3^Department of Respiratory and Critical Care Medicine, West China Medical School/West China Hospital, Sichuan University, Chengdu, China; ^4^Department of Targeted Tracer Research and Development Laboratory, West China Hospital, Sichuan University, Chengdu, China

**Keywords:** glucocorticoids, postoperative neurocognitive disorders, adult patients, surgery, meta-analysis

## Abstract

**Background:**

Postoperative neurocognitive disorders (PNDs) is common among surgical patients, however, the effect of glucocorticoids for preventing PNDs is not clear. This review aims to evaluate the effect of glucocorticoids on the incidence of PNDs in adult patients undergoing surgery.

**Methods:**

The databases of PubMed/Medline, Embase, the Cochrane Library, and Web of science were searched for all available randomized controlled trials (RCTs) from inception to April 30, 2022. RCTs comparing the effect of glucocorticoids with placebo on the incidence of PNDs in adult surgical patients (≥18 years old) were eligible. Subgroup analyses and meta-regressions were performed to evaluate sources of clinical heterogeneity. The level of certainty for main outcomes were assessed by the Grading of Recommendations Assessment, Development and Evaluation (GRADE) methodology.

**Results:**

Eleven trials with a total of 10,703 patients were identified. Compared with the control group, glucocorticoids did not reduce the incidence of PNDs (RR: 0.84, 95% CI: 0.67 to 1.06, *P* = 0.13, GRADE = moderate). Secondary analyses for primary outcome did not change the result. In addition, the length of ICU stay was decreased in glucocorticoids group (RR: −13.58, 95% CI: −26.37 to −0.80, *P* = 0.04, GRADE = low). However, there were no significant differences between groups with regards to the incidence of postoperative infection (RR: 0.94, 95% CI: 0.84 to 1.06, *P* = 0.30, GRADE = moderate), blood glucose level (RR: 1.05, 95% CI: −0.09 to 2.19, *P* = 0.07, GRADE = low), duration of mechanical ventilation (RR: −2.44, 95% CI: −5.47 to 0.59, *P* = 0.14, GRADE = low), length of hospital stay (RR: −0.09, 95% CI: −0.27 to 0.09, *P* = 0.33, GRADE = moderate) and 30-day mortality (RR: 0.86, 95% CI: 0.70 to 1.06, *P* = 0.16, GRADE = moderate).

**Conclusions:**

This meta-analysis suggests that perioperative administration of glucocorticoids may not reduce the incidence of PNDs after surgery. The effect of glucocorticoids on decreased length of ICU stay needs further researches. Future high-quality trials using acknowledged criteria and validated diagnostic tools are needed to determine the influence of glucocorticoids on long-term PNDs.

**Systematic Review Registration:**

https://www.crd.york.ac.uk/prospero/display_record.php?ID=CRD42022302262, identifier: CRD42022302262.

## Introduction

Postoperative neurocognitive disorders (PNDs) is an overarching term that includes postoperative delirium and postoperative cognitive dysfunction (POCD) (Vacas et al., 2021). According to the Perioperative Cognition Nomenclature Working Group in 2018 (Evered et al., 2018), postoperative delirium is an acute state of cognitive impairment occurring within days after surgery and up to 1 week or until discharge, while POCD is a prolonged cognitive decline usually detected between 30 days and 12 months postoperatively. It has been reported that postoperative delirium occurred in 10–60% of elderly surgical patients, varying by surgical procedures (American Geriatrics Society Expert Panel on Postoperative Delirium in Older Adults, 2015), and the incidence of POCD is approximately 25–40% (Wei et al., 2019). Old age, low educational levels, poor preoperative cognitive function, perioperative pain and complicated surgery process are thought to be risk factors of PNDs (Xie and Shen, 2018; Evered et al., 2020; O'Gara et al., 2021). PNDs are the very common and severe postoperative neurological complications with poor outcomes, including increasing the length of hospital stay, mortality, and the risk of long-term cognitive impairment. These would cause significant clinical, social, and financial burdens on the patients and their communities (Monk et al., 2008; Inouye et al., 2014; Boone et al., 2020).

Improving cognitive outcome after surgery, therefore, is an important objective for anesthesiologists and surgeons. To date, there have been no compelling pharmacologic interventions to limit the incidence or severity of PNDs (Mahanna-Gabrielli et al., 2019; Deemer et al., 2020). Dexmedetomidine, an anesthetic agent with neural anti-inflammatory effects, has been found to show promise for PNDs prevention (Lee et al., 2018; Likhvantsev et al., 2021). However, it has common side effects such as bradycardia and hypotension (Wu et al., 2018; Shi et al., 2020; Zhao et al., 2020), and the evidence to support this effect is limited (Sanders et al., 2021). For non-pharmacologic approaches, cognitive prehabilitation, physical activity, and management of hypertension and diabetes seem to be effective to improve cognitive function (Wang et al., 2020; Humeidan et al., 2021), but there is still a gap in their integration into pathways of care for patients (Vlisides et al., 2019; Deiner et al., 2020).

Proposed potential mechanisms for PNDs, including mitochondrial dysfunction, oxidative stress (Netto et al., 2018), synaptic damage (Xiao et al., 2018), and neurotrophic support impairment (Fan et al., 2016) are speculative, among which neuroinflammation is the most significantly concerned (Luo et al., 2019). It has been reported that surgery and anesthesia could lead the peripheral immune system to produce pro-inflammatory signals (Balusu et al., 2016; Noll et al., 2017). These inflammatory mediators could transfer into the brain through paraventricular areas of the blood-brain barrier (BBB) and stimulate microglia to produce proinflammatory factors, destroying synapses and neurons, thus causing neurotoxic symptoms and cognitive disorders (Lim et al., 2013; Liu and Yin, 2018).

Glucocorticoids are commonly used in the perioperative period to attenuate the inflammatory response (Holte and Kehlet, 2002; Lunn and Kehlet, 2013). And they can alleviate the inflammation by inhibiting prostaglandin production (Rhen and Cidlowski, 2005), activating endothelial nitric oxide synthetase (Hafezi-Moghadam et al., 2002), and decreasing the stability of mRNA for genes for inflammatory proteins (Gille et al., 2001; Lasa et al., 2002; Saklatvala et al., 2003). Evaluating whether the perioperative administration of glucocorticoids is helpful in preventing cognitive decline could promote targeted preventive and therapeutic interventions. Therefore, in recent years several studies have investigated the efficacy of glucocorticoids on cognitive disorders after anesthesia and surgery. However, their conclusions have been inconsistent. Qiao et al. (2015) and Valentin et al. (2016) investigated the effect of glucocorticoids on PNDs in elderly patients undergoing non-cardiac surgery. They found that the preventive administration of glucocorticoids could effectively reduce POCD. In contrast, Sauër et al. (2014) demonstrated the opposite result, showing that intraoperative administration of glucocorticoids did not reduce the incidence of delirium after cardiac surgery. Besides, Fang et al. (2014) studied the effect of glucocorticoids in patients suffering from facial spasms requiring microvascular decompression. They found that administering a higher dose of glucocorticoids increased the incidence of POCD in the early postoperative period. Therefore, we applied a systematic review and meta-analysis to explore the effect of perioperative glucocorticoids administration on the incidence of PNDs.

## Methods

This meta-analysis was conducted following the recommendations of Preferred Reporting Items for Systematic Reviews and Meta-Analyses (PRISMA) (Page et al., 2021). This study protocol was registered in PROSPERO database (CRD42022302262).

### Search Strategy

The databases of Pubmed/Medline, Embase, the Cochrane Library/Central, and Web of science were systematically searched for all relevant studies from inception to April 30, 2022. The references of included researches were also examined. According to the search strategy, both MeSH terms and free terms were used. The following keyword search terms were used: *glucocorticoids, cognitive disorders, delirium, and surgery*. The search strategy was given in the Supplementary Material.

### Study Selection Criteria

Studies restricted to randomized controlled trials (RCTs) in adult surgical patients (≥18 years old). All published full-article RCTs compared the effect of glucocorticoids with placebo or equal volume of normal saline (NS) on the incidence of PNDs were eligible for inclusion. Language restriction was not applied.

Pediatric surgery, non-intravenous administration of glucocorticoids, no available assessment tools, and animal experiments were excluded from this meta-analysis.

### Data Extraction

Data extraction and quality assessment were completed by two authors (XX and RG) independently. One author (XX) entered the information into the table and checked for consistency and completeness. Disagreements on data extraction and quality assessment were handled by discussion or reviewed by the third author (CC). The extracted data and information were as follows: first author, year of publication, surgery type, patient age, glucocorticoids type, timing and dose, control group, PNDs type, and cognitive assessment timing and methods. In addition, the following adverse events were extracted as well, including PNDs, infection, blood glucose level, duration of mechanical ventilation, length of ICU and hospital stay, and 30-day mortality.

### Quality Assessment

Quality assessment of included RCTs was performed according to the second version of the Cochrane risk-of-bias tool for RCTs (RoB 2.0) (Sterne et al., 2019). There are seven sections of this assessment, random sequence generation, allocation concealment, blinding of participants and personnel, blinding of outcome assessment, incomplete outcome data, selective reporting, and other biases. Each section was classified into the low, high, or unclear risk of bias.

### Endpoints

The primary endpoint of this meta-analysis was the incidence of PNDs. The secondary outcomes were the incidence of postoperative infection, blood glucose level, duration of mechanical ventilation, the length of ICU and hospital stay, and postoperative 30-day mortality.

### Statistical Analysis

For dichotomous data (incidence of PNDs, postoperative infection, and 30-day mortality), the Mantel-Haenszel method was used to combine outcomes and risk ratio (RR) with 95% confidence intervals (CI) were calculated. Concerning continuous variables (blood glucose level, duration of mechanical ventilation, and the length of ICU and hospital stay), the Inverse-Variance method was used, and mean difference (MD) or standardized mean difference (SMD) with 95% CI were calculated. The *I*^2^ statistics used to evaluate heterogeneity were divided into the following three levels (Melsen et al., 2014): low (*I*^2^ < 50%), moderate (*I*^2^ = 50–75%) and high (*I*^2^ > 75%). When the heterogeneity was low, we used fixed effects model to pooled the data; otherwise, we chose random effects model.

To find sources of heterogeneity, subgroup analyses were conducted according to the type of PNDs (postoperative delirium and POCD), the type of glucocorticoids, surgery, dose, and age. In subgroup analysis for the dose of glucocorticoids, the trials were stratified into three broad dose groups: low dose group if the total dose of glucocorticoid used was ≤ 30 mg prednisolone or equivalent, medium dose group if the total dose used was between 30 and 100 mg prednisolone or equivalent, and high dose group if the total dose used was >100 mg prednisolone or equivalent. These cut points were chosen according to clinical practice (Czock et al., 2005). For studies that used dexamethasone, methylprednisolone, or hydrocortisone, the total dose of glucocorticoid used was converted to an equivalent dose of prednisolone with similar glucocorticoid effect. The dose conversion factors for dexamethasone, methylprednisolone, and hydrocortisone to prednisolone were 6, 1.25, and 0.25, respectively (https://clincalc.com/corticosteroids/). In subgroup analysis for patient age, we calculated the mean age of the study population as the basis of classification. Besides, Hartung-Knapp adjusted meta-regressions were performed to find interactions between variables if subgroup analyses could not explain sources of heterogeneity.

Publication bias was assessed through visual inspection of funnel plots and Egger's test to evaluate the small-study effects. The influence of a potential publication bias on findings was explored by using the Duval and Tweedie trim-and-fill procedure. Sensitivity analyses were performed by excluding high-risk studies evaluated by RoB 2.0 or omitting one study each time to detect robustness of the pooled results. Finally, the level of certainty for main outcomes were assessed by the Grading of Recommendations Assessment, Development and Evaluation (GRADE) methodology (Langendam et al., 2013). *P* < 0.05 was considered statistically significant for all tests. All data analyses were performed by Revman 5.3. and Stata 16.

## Results

### Study Characteristics

**Figure 1:**
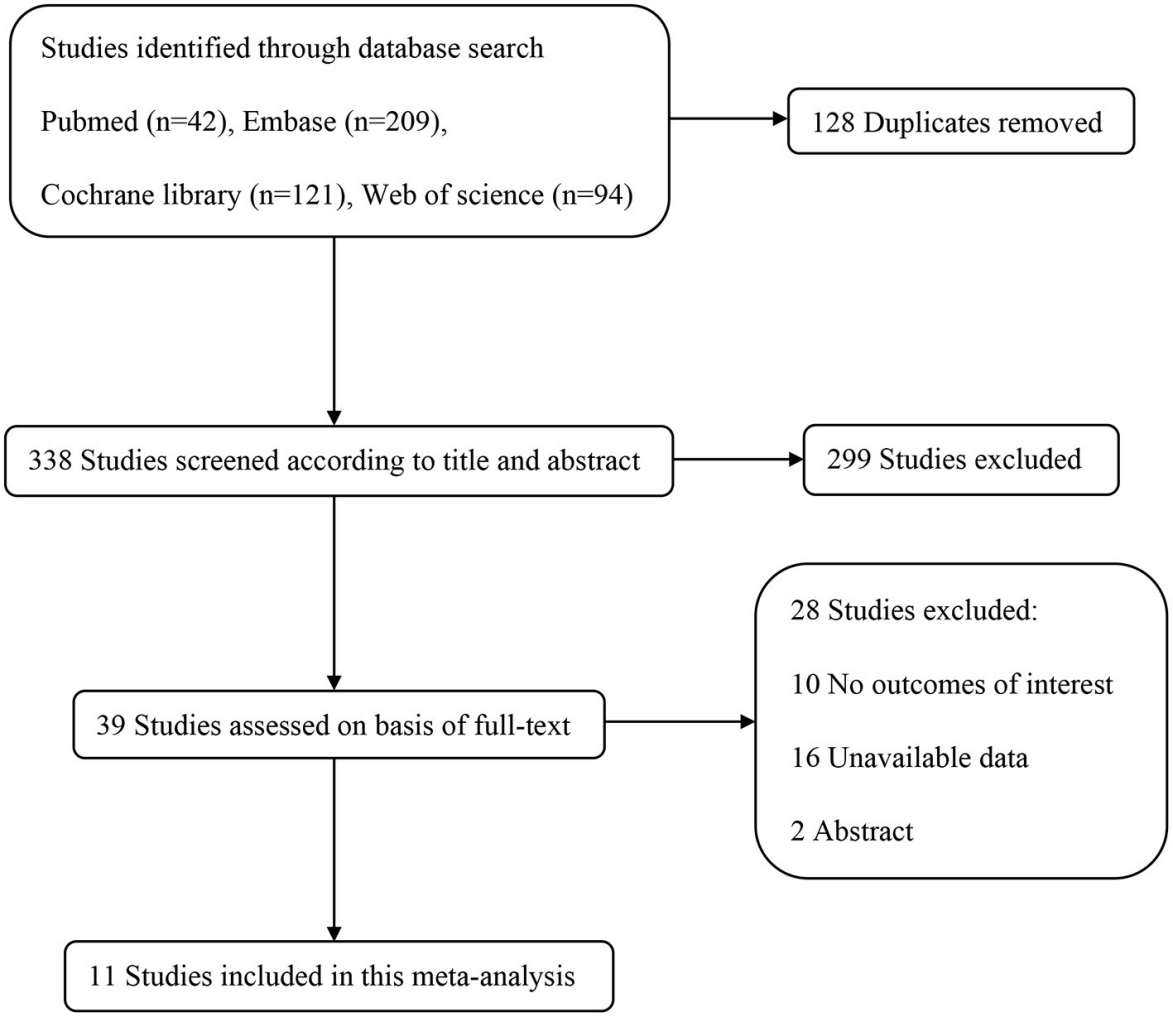
Flow chart of search strategy to identify the eligible randomized controlled trials.

**Figure 2:**
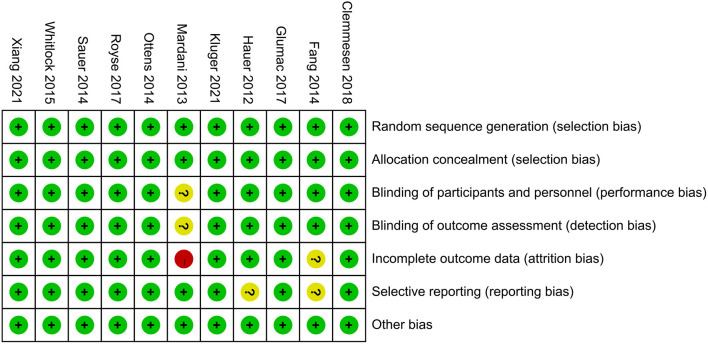
Risk of bias assessment was undertaken for each included trial according to the Cochrane Risk of Bias Methods.

According to the search strategy, a total of 466 trials were identified. Among them, 128 studies were removed due to duplication, and the other 327 studies were excluded based on inclusion and exclusion criteria. Ultimately, 11 RCTs, including 10,703 patients, were included in this meta-analysis. The selection process flow chart was shown in Figure 1, and methodological quality assessment was conducted according to the RoB 2.0, and the result was summarized in Figure 2. The major characters of these eligible studies were extracted and presented in Table 1. Seven trials were cardiac surgery (Hauer et al., 2012; Mardani and Bigdelian, 2013; Ottens et al., 2014; Sauër et al., 2014; Whitlock et al., 2015; Glumac et al., 2017; Royse et al., 2017), one trial was neurologic surgery (Fang et al., 2014), and three trials were non-cardiac, non-neurologic surgery, including one laparoscopic gastrointestinal surgery (Xiang et al., 2022) and two hip fracture surgery (Clemmesen et al., 2018; Kluger et al., 2021). Furthermore, six studies used dexamethasone as intervention (Mardani and Bigdelian, 2013; Fang et al., 2014; Ottens et al., 2014; Sauër et al., 2014; Glumac et al., 2017; Kluger et al., 2021), four studies used methylprednisolone (Whitlock et al., 2015; Royse et al., 2017; Clemmesen et al., 2018; Xiang et al., 2022), and one study used hydrocortisone (Hauer et al., 2012). All of the included studies used normal saline as a placebo. Besides, eight studies investigated postoperative delirium (Hauer et al., 2012; Mardani and Bigdelian, 2013; Sauër et al., 2014; Whitlock et al., 2015; Royse et al., 2017; Clemmesen et al., 2018; Kluger et al., 2021; Xiang et al., 2022) while the other three trials investigated POCD (Fang et al., 2014; Ottens et al., 2014; Glumac et al., 2017). The timing and dose of glucocorticoids administration and cognitive assessment methods were varied between included studies.

**Table 1:** Characteristics of the 11 included trails.

**First author**	**Year**	**Number of people (*n*)**	**Age (Y)**	**Surgery type**	**Glucocorticoids type**	**Doses, time and duration of intervention**	**Control**	**PNDs type**	**Assessment time**	**Assessment methods**
Mardani	2013	DEX: 43	DEX: 64.55 ± 11.10	Cardiac surgery	DEX	8 mg before surgery and 8 mg every 8 h for the first three POD	NS	Delirium	PROD and POD1–3	MMSE
		NS: 50	NS: 60.04 ± 12.77							
Sauer	2014	DEX: 367	DEX: 67 ± 12	Cardiac surgery	DEX	1 mg/kg (maximum 100 mg) at anesthetic induction	NS	Delirium	POD1–4	CAM-ICU, CAM
		NS: 370	NS: 66 ± 12							
Kluger	2021	DEX: 40	DEX: 81.4 ± 7.2	Hip fracture	DEX	20 mg before surgery	NS	Delirium	POD1–3	4AT
		NS: 39	NS: 81.4 ± 8.9							
Fang	2014	DEX-1: 320	DEX-1: 48.9 ± 5.35	Microvascular decompression	DEX	0.1 mg/kg or 0.2 mg/kg before anesthesia	NS	POCD	PROD1 and POD5	A battery of tests
		DEX-2: 315	DEX-2: 48.0 ± 5.60							
		NS: 319	NS: 48.0 ± 5.77							
Ottens	2014	DEX: 140	DEX: 63.4 ± 12.3	Cardiac surgery	DEX	1 mg/kg (maximum 100 mg) after anesthetic induction	NS	POCD	PROD1 and POD30 and 12 months	A battery of tests
		NS: 138	NS: 65.4 ± 11.5							
Glumac	2017	DEX: 80	DEX: 63.7 ± 9.0	Cardiac surgery	DEX	0.1 mg/kg 10 h before surgery	NS	POCD	PROD2 and POD6	A battery of tests
		NS: 81	NS: 64.2 ± 9.4							
Whitlock	2015	MET: 3755	MET: 67.5 ± 13.6	Cardiac surgery	MET	250 mg at anesthetic induction and 250 mg at initiation of CPB	NS	Delirium	POD3	CAM
		NS: 3752	NS: 67.3 ± 13.8							
Royse	2017	MET: 250	MET: 73.4 ± 10.5	Cardiac surgery	MET	250 mg at anesthetic induction and 250 mg at initiation of CPB	NS	Delirium	POD1–3	CAM-ICU
		NS: 248	NS: 74.3 ± 9.3							
Clemmesen	2018	MET: 59	MET: 79 ± 8	Hip fracture	MET	125mg before surgery	NS	Delirium	POD1–3	CAM-S
		NS: 58	NS: 81 ± 9							
Xiang	2021	MET: 84	MET: 71 (68–74)	Laparoscopic gastrointestinal surgery	MET	2 mg/kg of before surgery	NS	Delirium	POD1–5	CAM
		NS: 84	NS: 70 (68–73)							
Hauer	2012	HYD: 56	HYD: 69.3 ± 8.9	Cardiac surgery	HYD	100 mg over 10 min before anesthesia, and 10 mg/h on POD 1, 5 mg/h on POD 2, 3 ×20 mg on POD 3, 3 ×10 mg on POD 4	NS	ACD/delirium	POD1	DSM-IV
		NS: 55	NS: 68.0 ± 8.3							

*Date presented as mean ± SD or median (interquartile range)*.

*DEX, dexamethasone; MET, methylprednisolone; HYD, hydrocortisone; NS, normal saline; CPB, cardiopulmonary bypass; POCD, postoperative cognitive dysfunction; PROD, pre-operative day; POD, post-operative day; MMSE, mini-mental state examination; CAM, confusion assessment method; CAM-ICU, CAM for intensive care unit; CAM-S, confusion assessment method-short; 4AT, arousal, attention, abbreviated Mental Test–4, acute change; ACD, acute postoperative cognitive dysfunction; DSM-IV, Diagnostic and Statistical Manual of Mental Disorders, Fourth Revision*.

### Primary Outcome

**Figure 3:**
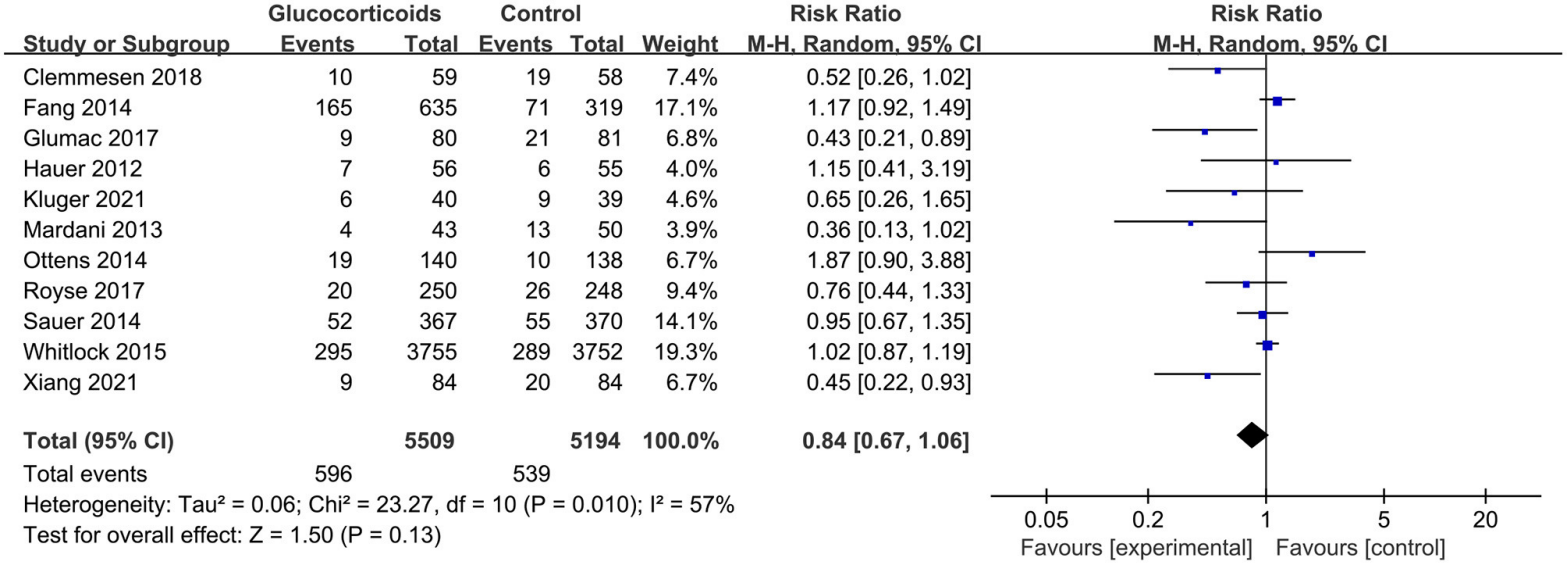
Forest plot for the incidence of PNDs.

**Figure 4:**
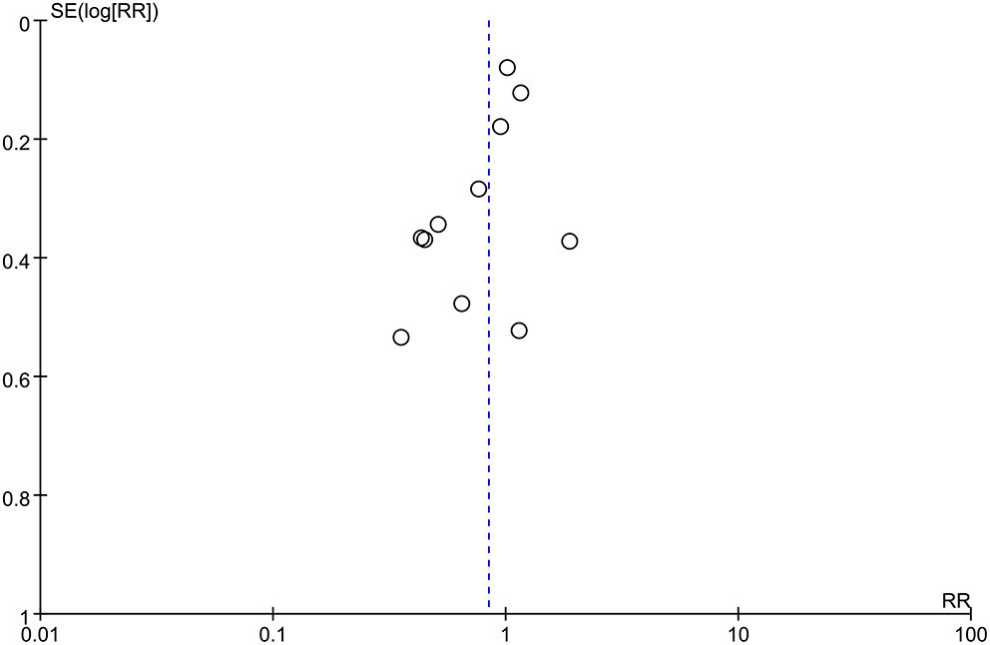
Funnel plot of the primary outcome (the incidence of PNDs).

The overall pooled result showed that glucocorticoids did not decrease the incidence of PNDs compared to the controls (RR: 0.84, 95% CI: 0.67 to 1.06, *P* = 0.13, *I*^2^ = 57%) (Figure 3). Sensitivity analyses were performed by excluding the high-risk study (Mardani and Bigdelian, 2013) or omitting one study each time from included studies, and the pooled result was still robust (Supplementary Figure 1). Meanwhile, no significant publication bias was evidenced by visual inspection of funnel plot (Figure 4) and Egger's test (Supplementary Table 1) for the effect of glucocorticoid administration on PNDs.

**Figure 5:**
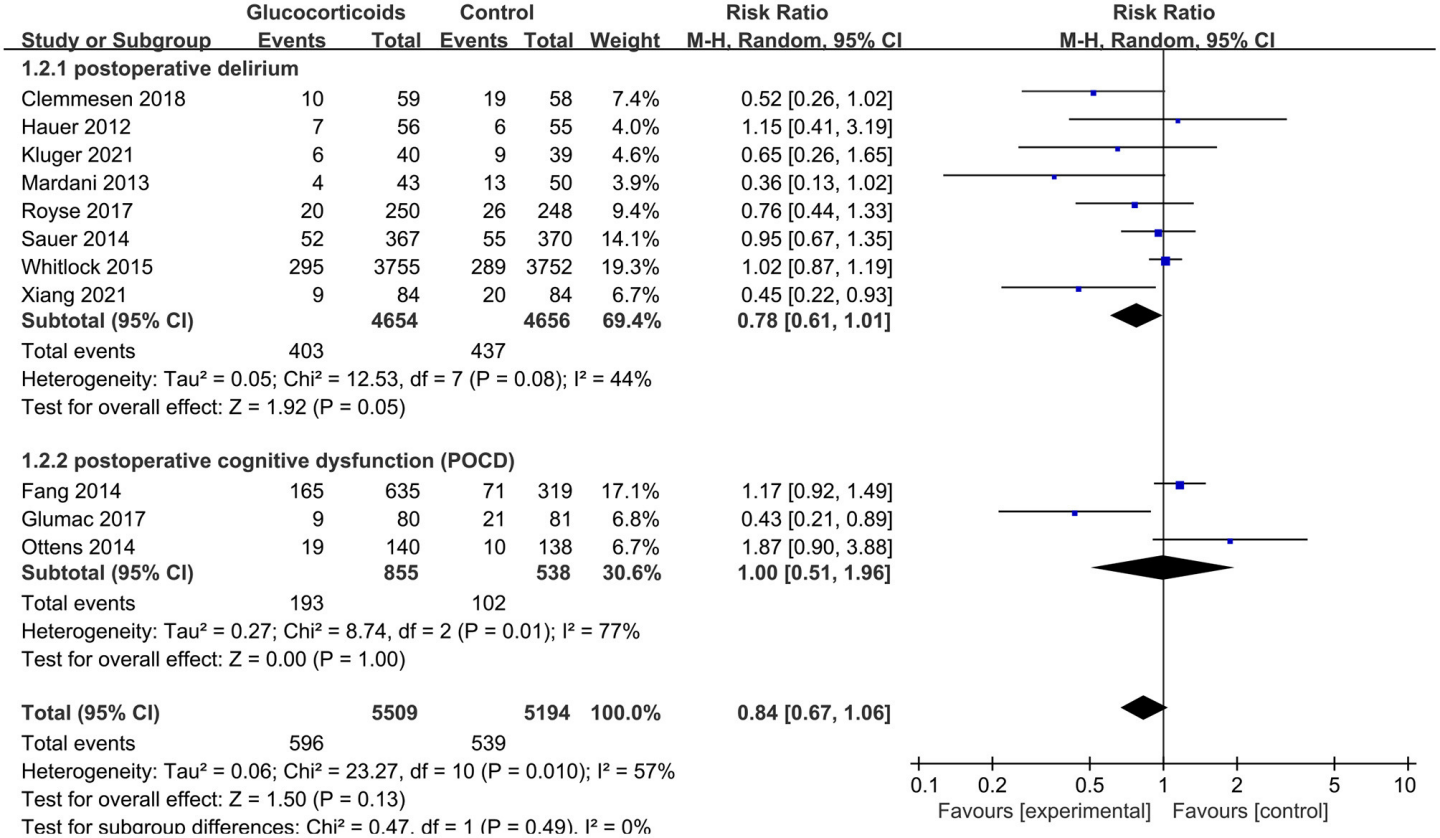
Subgroup analysis of the incidence of PNDs for the type of PNDs.

**Figure 6:**
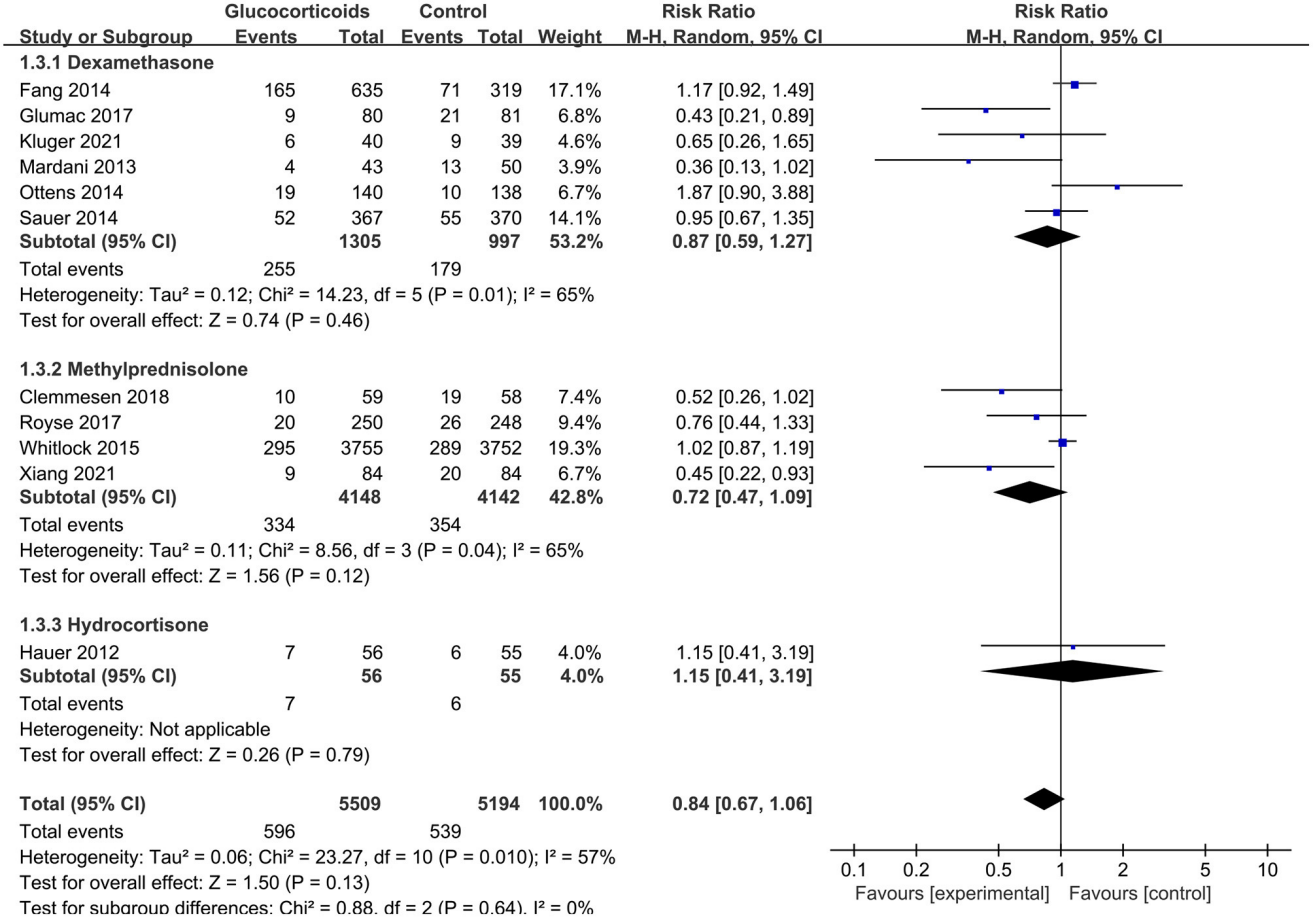
Subgroup analysis of the incidence of PNDs for the type of glucocorticoids.

Similarly, the finding was consistent in subgroup analyses between postoperative delirium (RR: 0.78, 95% CI: 0.61 to 1.01, *P* = 0.05, *I*^2^ = 44%) and POCD (RR: 1.00, 95% CI: 0.51 to 1.96, *P* = 1.00, *I*^2^ = 77%) (Figure 5), between dexamethasone (RR: 0.87, 95% CI: 0.59 to 1.27, *P* = 0.46, *I*^2^ = 65%), methylprednisolone (RR: 0.72, 95% CI: 0.47 to 1.09, *P* = 0.12, *I*^2^ = 65%) and hydrocortisone (Figure 6). However, subgroup analyses for glucocorticoids dose, surgery type and patient age showed the inconsistent results. There were significant differences in medium dose group (RR: 0.49, 95% CI: 0.33 to 0.73, *P* = 0.0005, *I*^2^ = 0%) (Supplementary Figure 2), non-cardiac, non-neurologic surgery group (RR: 0.52, 95% CI: 0.33 to 0.80, *P* = 0.003, *I*^2^ = 0%) (Supplementary Figure 3) and mean age ≥70 years group (RR: 0.60, 95% CI: 0.43 to 0.85, *P* = 0.004, *I*^2^ = 0%) (Supplementary Figure 4). However, further meta-regressions showed that when glucocorticoids type, surgery type, patient age, and their interactions were entered as covariates in models, there were no significant differences between glucocorticoid group and placebo group on the incidence of PNDs (Supplementary Table 2).

### Secondary Outcomes

**Figure 7:**
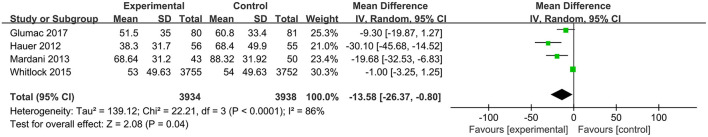
Forest plot of length of ICU stay.

**Figure 8:**
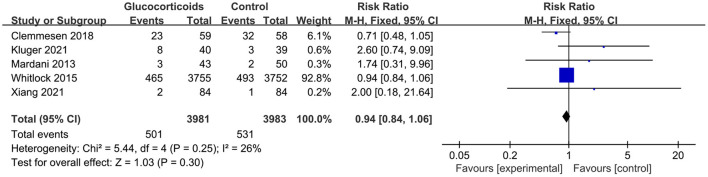
Forest plot of postoperative injection.

**Figure 9:**
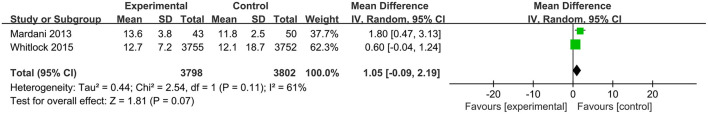
Forest plot of postoperative blood glucose level.

**Figure 10:**
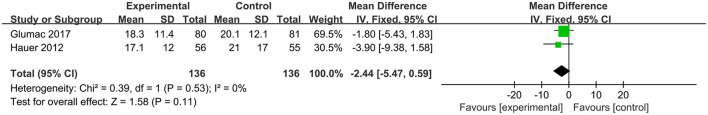
Forest plot of duration of mechanical ventilation.

**Figure 11:**
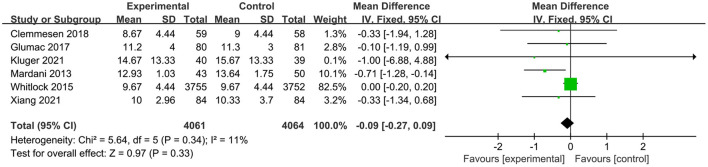
Forest plot of length of hospital stay.

**Figure 12:**
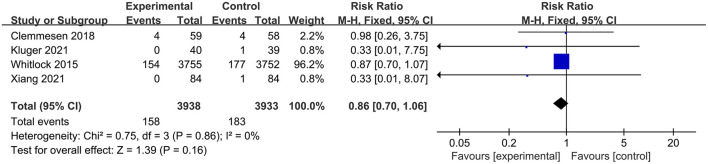
Forest plot of postoperative 30-day mortality.

Of the 11 studies included in this meta-analysis, four studies (Hauer et al., 2012; Mardani and Bigdelian, 2013; Whitlock et al., 2015; Glumac et al., 2017) compared the length of ICU stay between groups and the glucocorticoid group significantly reduced the length stay in ICU (RR: −13.58, 95% CI: −26.37 to −0.80, *P* = 0.04, *I*^2^ = 86%) (Figure 7). However, there were no significant differences in postoperative infection [five trials (Mardani and Bigdelian, 2013; Whitlock et al., 2015; Clemmesen et al., 2018; Kluger et al., 2021; Xiang et al., 2022); RR: 0.94, 95% CI: 0.84 to 1.06, *P* = 0.30, *I*^2^ = 26%] (Figure 8), blood glucose level [two trials (Mardani and Bigdelian, 2013; Whitlock et al., 2015); RR: 1.05, 95% CI: −0.09 to 2.19, *P* = 0.07, *I*^2^ = 61%] (Figure 9), duration of mechanical ventilation [two trials (Hauer et al., 2012; Glumac et al., 2017); RR: −2.44, 95% CI: −5.47 to 0.59, *P* = 0.14, *I*^2^ = 0%] (Figure 10), length of hospital stay [six trials (Mardani and Bigdelian, 2013; Whitlock et al., 2015; Glumac et al., 2017; Clemmesen et al., 2018; Kluger et al., 2021; Xiang et al., 2022); RR: −0.09, 95% CI: −0.27 to 0.09, *P* = 0.33, *I*^2^ = 11%] (Figure 11) and 30-day mortality [four trials (Whitlock et al., 2015; Clemmesen et al., 2018; Kluger et al., 2021; Xiang et al., 2022); RR: 0.86, 95% CI: 0.70 to 1.06, *P* = 0.16, *I*^2^ = 0%] (Figure 12).

### Level of Certainty for Outcomes (GRADE)

Basing on GRADE framework, we evaluated the level of certainty for our main outcomes. The quality of these outcomes varied from low to moderate and the detailed information were shown in Table 2.

**Table 2:** GRADE evidence for main outcomes.

**Outcomes (N RCTs)**	**Risk of bias**	**Inconsistency**	**Indirectness**	**Imprecision**	**Publication bias**	**Quality**
PNDs (11)	Not serious	No serious	Not serious	Serious^b^	Not detected	Moderate
Infection (5)	Not serious	Not serious	Not serious	Not serious	Strongly suspected^f^	Moderate
Blood glucose level (2)	Not serious	Not serious	Not serious	Serious^c^	Strongly suspected^f^	Low
Duration of mechanical ventilation (2)	Not serious	Not serious	Not serious	Serious^d^	Strongly suspected^f^	Low
Length of ICU stay (4)	Not serious	Serious^a^	Not serious	Not serious	Strongly suspected^f^	Low
Length of hospital stay (6)	Not serious	Not serious	Not serious	Not serious	Strongly suspected^f^	Moderate
30-day mortality (4)	Not serious	Not serious	Not serious	Serious^e^	Not detected	Moderate

*PNDs, postoperative neurocognitive disorders*.

^a^*Inconsistency due to significant statistical heterogeneity*.

^b^*Confidence intervals range from showing reduced incidence of PNDs associated with use of glucocorticoids to showing no clinically significant difference*.

^c^*Confidence intervals range from showing increased blood glucose level associated with use of glucocorticoids to showing no clinically significant difference*.

^d^*The number of patients included in this outcome was less than the optimal information size (OIS)*.

^e^*Confidence intervals range from showing decreased 30-day mortality associated with use of glucocorticoids to showing no clinically significant difference*.

^f^*Publication bias was suspected because the outcomes in the Dexamethasone for Cardiac Surgery (DECS) trial (Dieleman et al., 2012) were different and not included here*.

## Discussion

This meta-analysis suggests that perioperative glucocorticoids administration does not reduce the incidence of PNDs. Subgroup analyses and meta-regressions considering potential variables such as PNDs type, glucocorticoids type, dose, surgery type and patient age remained no difference in the outcome. Besides, glucocorticoids infusion was associated with a shorter length of ICU stay, while the incidence of postoperative infection, blood glucose level, duration of mechanical ventilation, length of hospital stay, and 30-day mortality did not differ significantly between groups.

Neuroinflammation has become a key hallmark of neurological complications including PNDs (Subramaniyan and Terrando, 2019). Perioperative glucocorticoid administration has been used in different surgical settings to counter the detrimental effect of inflammation induced by surgery and anesthesia (Awada et al., 2022). Several studies have shown that the preoperative administration of glucocorticoids reduces peripheral inflammatory markers in hepatic surgery (Orci et al., 2013; Richardson et al., 2014). However, the effect of glucocorticoids on PNDs was not observed despite pooling data for more than 10,000 randomized participants, with overall low risk of bias across studies in our review. Several reasons may account for this result. First, although excessive neuroinflammation leads to injury and death of neural elements, a large body of literatures have demonstrated that proper neuroinflammatory response can benefit outcomes of central nerve system (Yong et al., 2019). For example, neuroinflammation can promote neurogenesis (Ziv et al., 2006), facilitate axonal regeneration (David et al., 1990), and is critical for remyelination (Goldstein et al., 2016). It's crucial to suppress the excessive inflammation that mediates damage without inhibiting the repairment effect from preventing PNDs. Second, the genesis of PNDs is multifactorial that other factors except for neuroinflammation may play great roles in the development of neurocognitive deficit (Siddiqi et al., 2016). Third, prolonged exposure to high concentrations of glucocorticoids can be toxic to neural structures, especially the glucocorticoid receptor–rich hippocampus (Sapolsky, 2000).

To explore sources of heterogeneity, we performed subgroup analyses based on PNDs type and glucocorticoids type, and the outcome remained no difference. Although subgroup analyses about glucocorticoids dose, surgery type and patient age seemed significantly different, meta-regressions were further conducted to understand the interactions between these variables on our outcomes and the results changed to no difference. Here what should be noted was that the subgroup of age was classified according to the mean age values in studies, which might induce some misclassification of accurate age. In general, these secondary analyses suggest that the genesis of PNDs is multifactorial that only administration of glucocorticoids may not significantly reduce the incidence of neurocognitive disorders after surgery. Besides, the impact of patient, surgery, or other variables, both measured and unmeasured, on the PNDs development likely far outweighs the impact of glucocorticoids.

PNDs is a summarized term encompassing postoperative delirium, a most pronounced and acute postoperative form, and POCD which is described as a long-term neurocognitive impairment (Evered et al., 2018). Delirium and POCD previously were considered distinct entities, but recent data has suggested an underlying relationship between them (Olotu, 2020). Several risk factors are common to both postoperative delirium and POCD, and animal studies raise the possibility that neuroinflammation may play a role in both of these states (Berger et al., 2018; Devinney et al., 2018; Daiello et al., 2019). Therefore, in this meta-analysis we choose PNDs as the endpoint which in other words, combining postoperative delirium and POCD in the composite outcome, is acceptable from perspective of pathogenesis.

The diagnosis of PNDs, especially POCD, is complex requiring neuropsychological tests which are varied in studies. A recent systematic review noted that in 274 existing studies of POCD, diagnosis was based on 259 different cognitive assessment tools (Borchers et al., 2021). Moreover, neuropsychological tests have been undertaken at variable time intervals after anesthesia and surgery. In 2018, Evered et al. (2018) clarified that postoperative delirium was defined as occurring in hospital and up to 1 week post-procedure or until discharge, while POCD persisted for more than 30 days but <12 months following anesthesia and surgery. However, in trials included in this meta-analysis, Fang et al. (2014) and Glumac et al. (2017) examined POCD in postoperative day 5 and 6, respectively, in which delirium is usually to be assessed; while only Ottens et al. (2014) examined POCD at 1 month after surgery. In summary, heterogeneities of assessment tools, diagnostic criteria, and follow-up time limit the interpretation of existing data surrounding PNDs. Besides, there were only three studies (Fang et al., 2014; Ottens et al., 2014; Glumac et al., 2017) examining POCD in our meta-analysis, and two of them (Fang et al., 2014; Glumac et al., 2017) assessing this entity in the early postoperative day. Thus, the effect of glucocorticoids on long-term PNDs is still unclear. PNDs is associated with long-term sequelae including ongoing impaired cognition, increased risk of dementia, increased mortality, and premature retirement from work (Steinmetz et al., 2009). Interventions to mitigate these sequelae may therefore provide clinical and economic benefit in the long run. Further trials are needed to assess POCD using uniform criteria and validated diagnostic tools and then evaluate the effects of glucocorticoids on the incidence of long-term PNDs.

In our meta-analysis, glucocorticoids significantly reduced the length of ICU stay. However, there were only four studies examining this outcome with high heterogeneity. Besides, it is still obscure whether administration of glucocorticoids could impact the risk of postoperative infection, blood glucose level, length of hospital stay, duration of mechanical ventilation, and 30-day mortality. In the Dexamethasone for Cardiac Surgery (DECS) trial, Dieleman et al. (2012) reported that intraoperative administration of dexamethasone for cardiac surgery was associated with higher postoperative glucose level, lower infection rate, decreased duration of mechanical ventilation, and reduced length of hospital stay. Because of high heterogeneity and limited studies in this meta-analysis, future high-quality researches are still needed to confirm these outcomes.

To our knowledge, this is the first systematic review and meta-analysis to comprehensively evaluate effects of glucocorticoids on PNDs. A meta-analysis by Li et al. (2019) studied effects of dexamethasone on postoperative cognitive dysfunction and delirium in adults following general anesthesia, which did not take other types of glucocorticoids into consideration. Another systematic review and meta-analysis performed by Liu et al. (2021) was about effects of glucocorticoids on postoperative delirium in adult patients undergoing cardiac surgery. Similarly, POCD, another form of PNDs mentioned above, and non-cardiac surgery patients were not included in this study. In our review, we thought through PNDs type, glucocorticoids type, and surgery type to make conclusions as rigorous as possible.

There are still several potential limitations in this meta-analysis. First, we studied three types of glucocorticoids and two types of PNDs in cardiac, neurologic, and non-cardiac non-neurologic surgery, so there was potential heterogeneity such as methods of diagnosis and dosages of glucocorticoids, which may affect the precision and reliability of the results. Second, most of these included studies excluded patients with pre-existing cognitive impairment, and children were also excluded from this meta-analysis. Therefore, the extrapolation of this meta-analysis was limited. Third, some studies that contained our interested second outcomes, but not PNDs data, were excluded, thus influencing this meta-analysis's completeness of secondary outcomes. Further, more structured and standardized perioperative glucocorticoids protocols and uniform definition and assessment tools of PNDs may be necessary to accurately evaluate the effect of glucocorticoids on PNDs.

## Conclusion

In summary, our findings suggest that perioperative administration of glucocorticoids does not reduce the incidence of PNDs, regardless of PNDs type, glucocorticoids type, dose, surgery type and patient age. The effect of glucocorticoids on decreased length of ICU stay needs further researches. Future high-quality trials using acknowledged criteria and validated diagnostic tools are needed to determine the influence of glucocorticoids on long-term PNDs.

## Data Availability Statement

The original contributions presented in the study are included in the article/Supplementary Material, further inquiries can be directed to the corresponding author/s.

## Author Contributions

XX and RG wrote the manuscript and collected the data. XX, RG, TZ, and CC chose the topic. HC, XZ, and XC contributed to the conception. CZ and CL searched the literature. XX, RG, HC, XZ, and XC analyzed the data. TZ and CC made final decisions. All authors have read and approved the final manuscript.

## Funding

This work was supported by the National Natural Science Foundation of China (Nos. 82171185 and 81870858 to CC); The National Key R&D Program of China (No. 2018YFC2001800 to TZ) and the National Natural Science Foundation of China (No. 81671062 to TZ); China Postdoctoral Science Foundation (Grant No. 2020M673234 to RG), Post-doctoral Research Project, West China Hospital, Sichuan University (Grant No. 2020HXBH022 to RG).

## Conflict of Interest

The authors declare that the research was conducted in the absence of any commercial or financial relationships that could be construed as a potential conflict of interest.

## Publisher's Note

All claims expressed in this article are solely those of the authors and do not necessarily represent those of their affiliated organizations, or those of the publisher, the editors and the reviewers. Any product that may be evaluated in this article, or claim that may be made by its manufacturer, is not guaranteed or endorsed by the publisher.

## Supplementary Material

Supplementary Material Search strategy.

Supplementary Figure 1 Sensitivity analysis of the incidence of PNDs by excluding each study individually.

Supplementary Figure 2 Subgroup analysis of the incidence of PNDs for the dose of glucocorticoids.

Supplementary Figure 3 Subgroup analysis of the incidence of PNDs for the type of surgery.

Supplementary Figure 4 Subgroup analysis of the incidence of PNDs based on age classification.

Supplementary Table 1 Tests for publication bias.

Supplementary Table 2 Meta-regressions for the incidence of PNDs.

## References

[B1] American Geriatrics Society Expert Panel on Postoperative Delirium in Older Adults (2015). Postoperative delirium in older adults: best practice statement from the American Geriatrics Society. J. Am. Coll. Surg. 220, 136–148.e131. 10.1016/j.jamcollsurg.2014.10.019 25535170

[B2] Awada H. N. Steinthorsdottir K. J. Schultz N. A. Hillings,ø J. G. Larsen P. N. Jans Ø. . (2022). High-dose preoperative glucocorticoid for prevention of emergence and postoperative delirium in liver resection: a double-blinded randomized clinical trial substudy. Acta Anaesthesiol. Scand. 10.1111/aas.14057. [Epub ahead of print]. 35325467 PMC9320957

[B3] Balusu S. Van Wonterghem E. De Rycke R. Raemdonck K. Stremersch S. Gevaert K. . (2016). Identification of a novel mechanism of blood-brain communication during peripheral inflammation via choroid plexus-derived extracellular vesicles. EMBO Mol. Med. 8, 1162–1183. 10.15252/emmm.201606271 27596437 PMC5048366

[B4] Berger M. Terrando N. Smith S. K. Browndyke J. N. Newman M. F. Mathew J. P. (2018). Neurocognitive function after cardiac surgery: from phenotypes to mechanisms. Anesthesiology 129, 829–851. 10.1097/aln.0000000000002194 29621031 PMC6148379

[B5] Boone M. D. Sites B. von Recklinghausen F. M. Mueller A. Taenzer A. H. Shaefi S. (2020). Economic burden of postoperative neurocognitive disorders among US medicare patients. JAMA Netw. Open 3, e208931. 10.1001/jamanetworkopen.2020.8931 32735336 PMC7395237

[B6] Borchers F. Spies C. D. Feinkohl I. Brockhaus W. R. Kraft A. Kozma P. . (2021). Methodology of measuring postoperative cognitive dysfunction: a systematic review. Br. J. Anaesth. 126, 1119–1127. 10.1016/j.bja.2021.01.035 33820655

[B7] Clemmesen C. G. Lunn T. H. Kristensen M. T. Palm H. Foss N. B. (2018). Effect of a single pre-operative 125 mg dose of methylprednisolone on postoperative delirium in hip fracture patients; a randomised, double-blind, placebo-controlled trial. Anaesthesia 73, 1353–1360. 10.1111/anae.14406 30151823

[B8] Czock D. Keller F. Rasche F. M. Häussler U. (2005). Pharmacokinetics and pharmacodynamics of systemically administered glucocorticoids. Clin. Pharmacokinet. 44, 61–98. 10.2165/00003088-200544010-00003 15634032

[B9] Daiello L. A. Racine A. M. Yun Gou R. Marcantonio E. R. Xie Z. Kunze L. J. . (2019). Postoperative delirium and postoperative cognitive dysfunction: overlap and divergence. Anesthesiology 131, 477–491. 10.1097/aln.0000000000002729 31166241 PMC6692220

[B10] David S. Bouchard C. Tsatas O. Giftochristos N. (1990). Macrophages can modify the nonpermissive nature of the adult mammalian central nervous system. Neuron 5, 463–469. 10.1016/0896-6273(90)90085-t 2206534

[B11] Deemer K. Zjadewicz K. Fiest K. Oviatt S. Parsons M. Myhre B. . (2020). Effect of early cognitive interventions on delirium in critically ill patients: a systematic review. Can. J. Anaesth. 67, 1016–1034. 10.1007/s12630-020-01670-z 32333291 PMC7222136

[B12] Deiner S. Fleisher L. A. Leung J. M. Peden C. Miller T. Neuman M. D. (2020). Adherence to recommended practices for perioperative anesthesia care for older adults among US anesthesiologists: results from the ASA Committee on Geriatric Anesthesia-Perioperative Brain Health Initiative ASA member survey. Perioper. Med. 9, 6. 10.1186/s13741-020-0136-9 32123562 PMC7041201

[B13] Devinney M. J. Mathew J. P. Berger M. (2018). Postoperative delirium and postoperative cognitive dysfunction: two sides of the same coin? Anesthesiology 129, 389–391. 10.1097/aln.0000000000002338 29965817 PMC6092234

[B14] Dieleman J. M. Nierich A. P. Rosseel P. M. van der Maaten J. M. Hofland J. Diephuis J. C. . (2012). Intraoperative high-dose dexamethasone for cardiac surgery: a randomized controlled trial. JAMA 308, 1761–1767. 10.1001/jama.2012.14144 23117776

[B15] Evered L. Silbert B. Knopman D. S. Scott D. A. DeKosky S. T. Rasmussen L. S. . (2018). Recommendations for the nomenclature of cognitive change associated with anaesthesia and surgery-2018. Br. J. Anaesth. 121, 1005–1012. 10.1016/j.bja.2017.11.087 30336844 PMC7069032

[B16] Evered L. A. Vitug S. Scott D. A. Silbert B. (2020). Preoperative frailty predicts postoperative neurocognitive disorders after total hip joint replacement surgery. Anesth. Analg. 131, 1582–1588. 10.1213/ane.0000000000004893 33079882

[B17] Fan D. Li J. Zheng B. Hua L. Zuo Z. (2016). Enriched environment attenuates surgery-induced impairment of learning, memory, and neurogenesis possibly by preserving BDNF expression. Mol. Neurobiol. 53, 344–354. 10.1007/s12035-014-9013-1 25432890

[B18] Fang Q. Qian X. An J. Wen H. Cope D. K. Williams J. P. (2014). Higher dose dexamethasone increases early postoperative cognitive dysfunction. J. Neurosurg. Anesthesiol. 26, 220–225. 10.1097/ana.0000000000000024 24621831

[B19] Gille J. Reisinger K. Westphal-Varghese B. Kaufmann R. (2001). Decreased mRNA stability as a mechanism of glucocorticoid-mediated inhibition of vascular endothelial growth factor gene expression by cultured keratinocytes. J. Invest. Dermatol. 117, 1581–1587. 10.1046/j.0022-202x.2001.01573.x 11886526

[B20] Glumac S. Kardum G. Sodic L. Supe-Domic D. Karanovic N. (2017). Effects of dexamethasone on early cognitive decline after cardiac surgery: a randomised controlled trial. Eur. J. Anaesthesiol. 34, 776–784. 10.1097/eja.0000000000000647 28985195

[B21] Goldstein E. Z. Church J. S. Hesp Z. C. Popovich P. G. McTigue D. M. (2016). A silver lining of neuroinflammation: Beneficial effects on myelination. Exp. Neurol. 283(Pt B), 550–559. 10.1016/j.expneurol.2016.05.001 27151600

[B22] Hafezi-Moghadam A. Simoncini T. Yang Z. Limbourg F. P. Plumier J. C. Rebsamen M. C. . (2002). Acute cardiovascular protective effects of corticosteroids are mediated by non-transcriptional activation of endothelial nitric oxide synthase. Nat. Med. 8, 473–479. 10.1038/nm0502-473 11984591 PMC2668717

[B23] Hauer D. Weis F. Campolongo P. Schopp M. Beiras-Fernandez A. Strewe C. . (2012). Glucocorticoid-endocannabinoid interaction in cardiac surgical patients: relationship to early cognitive dysfunction and late depression. Rev. Neurosci. 23, 681–690. 10.1515/revneuro-2012-0058 23006898

[B24] Holte K. Kehlet H. (2002). Perioperative single-dose glucocorticoid administration: pathophysiologic effects and clinical implications. J. Am. Coll. Surg. 195, 694–712. 10.1016/s1072-7515(02)01491-6 12437261

[B25] Humeidan M. L. Reyes J. C. Mavarez-Martinez A. Roeth C. Nguyen C. M. Sheridan E. . (2021). Effect of cognitive prehabilitation on the incidence of postoperative delirium among older adults undergoing major noncardiac surgery: the neurobics randomized clinical trial. JAMA Surg. 156, 148–156. 10.1001/jamasurg.2020.4371 33175114 PMC7658803

[B26] Inouye S. K. Westendorp R. G. Saczynski J. S. (2014). Delirium in elderly people. Lancet 383, 911–922. 10.1016/s0140-6736(13)60688-1 23992774 PMC4120864

[B27] Kluger M. T. Skarin M. Collier J. Rice D. A. McNair P. J. Seow M. Y. . (2021). Steroids to reduce the impact on delirium (STRIDE): a double-blind, randomised, placebo-controlled feasibility trial of pre-operative dexamethasone in people with hip fracture. Anaesthesia 76, 1031–1041. 10.1111/anae.15465 33899214

[B28] Langendam M. W. Akl E. A. Dahm P. Glasziou P. Guyatt G. Schünemann H. J. (2013). Assessing and presenting summaries of evidence in Cochrane reviews. Syst. Rev. 2, 81. 10.1186/2046-4053-2-81 24059250 PMC3849859

[B29] Lasa M. Abraham S. M. Boucheron C. Saklatvala J. Clark A. R. (2002). Dexamethasone causes sustained expression of mitogen-activated protein kinase (MAPK) phosphatase 1 and phosphatase-mediated inhibition of MAPK p38. Mol. Cell. Biol. 22, 7802–7811. 10.1128/mcb.22.22.7802-7811.2002 12391149 PMC134716

[B30] Lee C. Lee C. H. Lee G. Lee M. Hwang J. (2018). The effect of the timing and dose of dexmedetomidine on postoperative delirium in elderly patients after laparoscopic major non-cardiac surgery: a double blind randomized controlled study. J. Clin. Anesth. 47, 27–32. 10.1016/j.jclinane.2018.03.007 29549829

[B31] Li L. Q. Wang C. Fang M. D. Xu H. Y. Lu H. L. Zhang H. Z. (2019). Effects of dexamethasone on post-operative cognitive dysfunction and delirium in adults following general anaesthesia: a meta-analysis of randomised controlled trials. BMC Anesthesiol. 19:113. 10.1186/s12871-019-0783-x 31253079 PMC6599229

[B32] Likhvantsev V. V. Landoni G. Grebenchikov O. A. Ovezov A. M. Skripkin Y. V. Lembo R. . (2021). Perioperative dexmedetomidine supplement decreases delirium incidence after adult cardiac surgery: a randomized, double-blind, controlled study. J. Cardiothorac. Vasc. Anesth. 35, 449–457. 10.1053/j.jvca.2020.02.035 32265083

[B33] Lim A. Krajina K. Marsland A. L. (2013). Peripheral inflammation and cognitive aging. Mod Trends Pharmacopsychiatry 28, 175–187. 10.1159/000346362 25224899

[B34] Liu W. Wang Y. Wang J. Shi J. Pan J. Wang D. (2021). Effects of glucocorticoids on postoperative delirium in adult patients undergoing cardiac surgery: a systematic review and meta-analysis. Clin. Ther. 43, 1608–1621. 10.1016/j.clinthera.2021.07.021 34548175

[B35] Liu Y. Yin Y. (2018). Emerging roles of immune cells in postoperative cognitive dysfunction. Mediators Inflamm. 2018, 6215350. 10.1155/2018/6215350 29670465 PMC5835271

[B36] Lunn T. H. Kehlet H. (2013). Perioperative glucocorticoids in hip and knee surgery – benefit vs. harm? A review of randomized clinical trials. Acta Anaesthesiol. Scand. 57, 823–834. 10.1111/aas.12115 23581549

[B37] Luo A. Yan J. Tang X. Zhao Y. Zhou B. Li S. (2019). Postoperative cognitive dysfunction in the aged: the collision of neuroinflammaging with perioperative neuroinflammation. Inflammopharmacology 27, 27–37. 10.1007/s10787-018-00559-0 30607668

[B38] Mahanna-Gabrielli E. Schenning K. J. Eriksson L. I. Browndyke J. N. Wright C. B. Culley D. J. . (2019). State of the clinical science of perioperative brain health: report from the American Society of Anesthesiologists Brain Health Initiative Summit 2018. Br. J. Anaesth. 123, 464–478. 10.1016/j.bja.2019.07.004 31439308 PMC6871269

[B39] Mardani D. Bigdelian H. (2013). Prophylaxis of dexamethasone protects patients from further post-operative delirium after cardiac surgery: a randomized trial. J. Res. Med. Sci. 18, 137–143. 23914217 PMC3724375

[B40] Melsen W. G. Bootsma M. C. Rovers M. M. Bonten M. J. (2014). The effects of clinical and statistical heterogeneity on the predictive values of results from meta-analyses. Clin. Microbiol. Infect. 20, 123–129. 10.1111/1469-0691.12494 24320992

[B41] Monk T. G. Weldon B. C. Garvan C. W. Dede D. E. van der Aa M. T. Heilman K. M. . (2008). Predictors of cognitive dysfunction after major noncardiac surgery. Anesthesiology 108, 18–30. 10.1097/01.anes.0000296071.19434.1e 18156878

[B42] Netto M. B. de Oliveira Junior A. N. Goldim M. Mathias K. Fileti M. E. da Rosa N. . (2018). Oxidative stress and mitochondrial dysfunction contributes to postoperative cognitive dysfunction in elderly rats. Brain Behav. Immun. 73, 661–669. 10.1016/j.bbi.2018.07.016 30041011

[B43] Noll F. Behnke J. Leiting S. Troidl K. Alves G. T. Müller-Redetzky H. . (2017). Self-extracellular RNA acts in synergy with exogenous danger signals to promote inflammation. PLoS ONE 12:e0190002. 10.1371/journal.pone.0190002 29261777 PMC5738100

[B44] O'Gara B. P. Gao L. Marcantonio E. R. Subramaniam B. (2021). Sleep, pain, and cognition: modifiable targets for optimal perioperative brain health. Anesthesiology 135, 1132–1152. 10.1097/aln.0000000000004046 34731233 PMC8578455

[B45] Olotu C. (2020). Postoperative neurocognitive disorders. Curr. Opin. Anaesthesiol. 33, 101–108. 10.1097/aco.0000000000000812 31764008

[B46] Orci L. A. Toso C. Mentha G. Morel P. Majno P. E. (2013). Systematic review and meta-analysis of the effect of perioperative steroids on ischaemia-reperfusion injury and surgical stress response in patients undergoing liver resection. Br. J. Surg. 100, 600–609. 10.1002/bjs.9035 23339056

[B47] Ottens T. H. Dieleman J. M. Sauër A. M. Peelen L. M. Nierich A. P. de Groot W. J. . (2014). Effects of dexamethasone on cognitive decline after cardiac surgery: a randomized clinical trial. Anesthesiology 121, 492–500. 10.1097/aln.0000000000000336 25225745

[B48] Page M. J. Moher D. Bossuyt P. M. Boutron I. Hoffmann T. C. Mulrow C. D. . (2021). PRISMA 2020 explanation and elaboration: updated guidance and exemplars for reporting systematic reviews. BMJ 372, n160. 10.1136/bmj.n160 33781993 PMC8005925

[B49] Qiao Y. Feng H. Zhao T. Yan H. Zhang H. Zhao X. (2015). Postoperative cognitive dysfunction after inhalational anesthesia in elderly patients undergoing major surgery: the influence of anesthetic technique, cerebral injury and systemic inflammation. BMC Anesthesiol. 15:154. 10.1186/s12871-015-0130-9 26497059 PMC4619426

[B50] Rhen T. Cidlowski J. A. (2005). Antiinflammatory action of glucocorticoids–new mechanisms for old drugs. N. Engl. J. Med. 353, 1711–1723. 10.1056/NEJMra050541 16236742

[B51] Richardson A. J. Laurence J. M. Lam V. W. (2014). Use of pre-operative steroids in liver resection: a systematic review and meta-analysis. HPB 16, 12–19. 10.1111/hpb.12066 23461716 PMC3892310

[B52] Royse C. F. Saager L. Whitlock R. Ou-Young J. Royse A. Vincent J. . (2017). Impact of methylprednisolone on postoperative quality of recovery and delirium in the steroids in cardiac surgery trial: a randomized, double-blind, placebo-controlled substudy. Anesthesiology 126, 223–233. 10.1097/aln.0000000000001433 27775998

[B53] Saklatvala J. Dean J. Clark A. (2003). Control of the expression of inflammatory response genes. Biochem. Soc. Symp. 70, 95–106. 10.1042/bss0700095 14587285

[B54] Sanders R. D. Wehrman J. Irons J. Dieleman J. Scott D. Shehabi Y. (2021). Meta-analysis of randomised controlled trials of perioperative dexmedetomidine to reduce delirium and mortality after cardiac surgery. Br. J. Anaesth. 127, e168–e170. 10.1016/j.bja.2021.08.009 34489090

[B55] Sapolsky R. M. (2000). Glucocorticoids and hippocampal atrophy in neuropsychiatric disorders. Arch. Gen. Psychiatry 57, 925–935. 10.1001/archpsyc.57.10.925 11015810

[B56] Sauër A. M. Slooter A. J. Veldhuijzen D. S. van Eijk M. M. Devlin J. W. van Dijk D. (2014). Intraoperative dexamethasone and delirium after cardiac surgery: a randomized clinical trial. Anesth Analg 119, 1046–1052. 10.1213/ane.0000000000000248 24810262

[B57] Shi H. Du X. Wu F. Hu Y. Xv Z. Mi W. (2020). Dexmedetomidine improves early postoperative neurocognitive disorder in elderly male patients undergoing thoracoscopic lobectomy. Exp. Ther. Med. 20, 3868–3877. 10.3892/etm.2020.9113 32855737 PMC7444346

[B58] Siddiqi N. Harrison J. K. Clegg A. Teale E. A. Young J. Taylor J. . (2016). Interventions for preventing delirium in hospitalised non-ICU patients. Cochrane Database Syst. Rev. 3, Cd005563. 10.1002/14651858.CD005563.pub3 26967259 PMC10431752

[B59] Steinmetz J. Christensen K. B. Lund T. Lohse N. Rasmussen L. S. (2009). Long-term consequences of postoperative cognitive dysfunction. Anesthesiology 110, 548–555. 10.1097/ALN.0b013e318195b569 19225398

[B60] Sterne J. A. C. Savović J. Page M. J. Elbers R. G. Blencowe N. S. Boutron I. . (2019). RoB 2: a revised tool for assessing risk of bias in randomised trials. BMJ 366, l4898. 10.1136/bmj.l4898 31462531

[B61] Subramaniyan S. Terrando N. (2019). Neuroinflammation and perioperative neurocognitive disorders. Anesth. Analg. 128, 781–788. 10.1213/ane.0000000000004053 30883423 PMC6437083

[B62] Vacas S. Cole D. J. Cannesson M. (2021). Cognitive decline associated with anesthesia and surgery in older patients. JAMA. 10.1001/jama.2021.4773. [Epub ahead of print]. 34338712 PMC8807795

[B63] Valentin L. S. Pereira V. F. Pietrobon R. S. Schmidt A. P. Oses J. P. Portela L. V. . (2016). Effects of single low dose of dexamethasone before noncardiac and nonneurologic surgery and general anesthesia on postoperative cognitive dysfunction – a phase III double blind, randomized clinical trial. PLoS ONE 11:e0152308. 10.1371/journal.pone.0152308 27152422 PMC4859565

[B64] Vlisides P. E. Das A. R. Thompson A. M. Kunkler B. Zierau M. Cantley M. J. . (2019). Home-based cognitive prehabilitation in older surgical patients: a feasibility study. J. Neurosurg. Anesthesiol. 31, 212–217. 10.1097/ana.0000000000000569 30557230 PMC6652226

[B65] Wang Y. Y. Yue J. R. Xie D. M. Carter P. Li Q. L. Gartaganis S. L. . (2020). Effect of the tailored, family-involved hospital elder life program on postoperative delirium and function in older adults: a randomized clinical trial. JAMA Intern. Med. 180, 17–25. 10.1001/jamainternmed.2019.4446 31633738 PMC6806427

[B66] Wei P. Yang F. Zheng Q. Tang W. Li J. (2019). The potential role of the NLRP3 inflammasome activation as a link between mitochondria ROS generation and neuroinflammation in postoperative cognitive dysfunction. Front. Cell. Neurosci. 13:73. 10.3389/fncel.2019.00073 30873011 PMC6401615

[B67] Whitlock R. P. Devereaux P. J. Teoh K. H. Lamy A. Vincent J. Pogue J. . (2015). Methylprednisolone in patients undergoing cardiopulmonary bypass (SIRS): a randomised, double-blind, placebo-controlled trial. Lancet 386, 1243–1253. 10.1016/s0140-6736(15)00273-1 26460660

[B68] Wu M. Liang Y. Dai Z. Wang S. (2018). Perioperative dexmedetomidine reduces delirium after cardiac surgery: a meta-analysis of randomized controlled trials. J. Clin. Anesth. 50, 33–42. 10.1016/j.jclinane.2018.06.045 29958125

[B69] Xiang X. B. Chen H. Wu Y. L. Wang K. Yue X. Cheng X. Q. (2022). The effect of preoperative methylprednisolone on postoperative delirium in older patients undergoing gastrointestinal surgery: a randomized, double-blind, placebo-controlled trial. J. Gerontol. A Biol. Sci. Med. Sci. 77, 517–523. 10.1093/gerona/glab248 34423832

[B70] Xiao J. Y. Xiong B. R. Zhang W. Zhou W. C. Yang H. Gao F. . (2018). PGE2-EP3 signaling exacerbates hippocampus-dependent cognitive impairment after laparotomy by reducing expression levels of hippocampal synaptic plasticity-related proteins in aged mice. CNS Neurosci. Ther. 24, 917–929. 10.1111/cns.12832 29488342 PMC6490074

[B71] Xie Z. Shen Y. (2018). New biomarkers of postoperative neurocognitive disorders. Nat. Rev. Neurol. 14, 320–321. 10.1038/s41582-018-0001-3 29622780

[B72] Yong H. Y. F. Rawji K. S. Ghorbani S. Xue M. Yong V. W. (2019). The benefits of neuroinflammation for the repair of the injured central nervous system. Cell. Mol. Immunol. 16, 540–546. 10.1038/s41423-019-0223-3 30874626 PMC6804643

[B73] Zhao W. Hu Y. Chen H. Wang X. Wang L. Wang Y. . (2020). The effect and optimal dosage of dexmedetomidine plus sufentanil for postoperative analgesia in elderly patients with postoperative delirium and early postoperative cognitive dysfunction: a single-center, prospective, randomized, double-blind, controlled trial. Front. Neurosci. 14:549516. 10.3389/fnins.2020.549516 33192244 PMC7645155

[B74] Ziv Y. Ron N. Butovsky O. Landa G. Sudai E. Greenberg N. . (2006). Immune cells contribute to the maintenance of neurogenesis and spatial learning abilities in adulthood. Nat. Neurosci. 9, 268–275. 10.1038/nn1629 16415867

