# Supplementary material for: Effects of Glucocorticoids on Postoperative Neurocognitive Disorders in Adult Patients: A Systematic Review and Meta-Analysis
Source: Front Aging Neurosci. 2022 Jun 30;14:939848. doi: 10.3389/fnagi.2022.939848 (PMC9284274; doi:10.3389/fnagi.2022.939848)
Supplement: Supplementary Material — Search strategy. [file Data_Sheet_1.ZIP › Supplementary Material/Supplementary Material Search Strategy.docx]

# Supplementary Material Search Strategy

# Part 1 – Glucocorticoids Terms

("Glucocorticoids"[Mesh]) OR (((((((((((Glucocorticoid*[Title/Abstract]) OR (Glucocorticoid Effect*[Title/Abstract])) OR (Predniso*[Title/Abstract])) OR (Methylprednisolone[Title/Abstract])) OR (Dexamethasone[Title/Abstract])) OR (Hydrocortisone[Title/Abstract])) OR (Triamcinolone[Title/Abstract])) OR (Betamethasone[Title/Abstract])) OR (Fluocinolone Acetonide[Title/Abstract])) OR (Corti*[Title/Abstract])) OR (Adrenal Cortex Hormone*[Title/Abstract]))

# Part 2 – PNDs Terms

("Neurocognitive Disorders"[Mesh]) OR (((((((((((Neurocognitive Disorder*[Title/Abstract]) OR (Cognitive Disorder*[Title/Abstract])) OR (Deliri*[Title/Abstract])) OR (Cognitive Dysfunction*[Title/Abstract])) OR (Cognitive Impairment*[Title/Abstract])) OR (Cognitive Decline*[Title/Abstract])) OR (Cognitive Deficit*[Title/Abstract])) OR (Cognitive Change*[Title/Abstract])) OR (Postoperative Cognitive Complication*[Title/Abstract])) OR (Postoperative Cognitive Dysfunction*[Title/Abstract])) OR (Neurocognitive Complication*[Title/Abstract]))

# Part 3 – Surgery Terms

("Surgical Procedures, Operative"[Mesh]) OR ((surger*[Title/Abstract]) OR (surgical[Title/Abstract]))

# Part 4 – Glucocorticoids and PNDs Terms combined

Part 1 AND Part 2

# Part 5 – Glucocorticoids, PNDs and Surgery Terms combined

Part 4 AND Part 3

# Part 6 – Random Controlled Trials limit applied to Part 5
