# Supplementary material for: Effects of Glucocorticoids on Postoperative Neurocognitive Disorders in Adult Patients: A Systematic Review and Meta-Analysis
Source: Front Aging Neurosci. 2022 Jun 30;14:939848. doi: 10.3389/fnagi.2022.939848 (PMC9284274; doi:10.3389/fnagi.2022.939848)
Supplement: Supplementary Material — Search strategy. [file Data_Sheet_1.ZIP › Supplementary Material/Supplementary Table 1.docx]

**Supplementary Table 1** Tests for publication bias

Egger's test

| Std_Eff | Coef. | Std. Err. | t | P>\|t\| | [95% Conf. Interval] | |
| --- | --- | --- | --- | --- | --- | --- |
| slope | .1780845 | .1252285 | 1.42 | 0.189 | -.1052021 | .4613711 |
| bias | -1.388543 | .6635017 | -2.09 | 0.066 | -2.889488 | .1124025 |
