# Supplementary material for: Effects of Glucocorticoids on Postoperative Neurocognitive Disorders in Adult Patients: A Systematic Review and Meta-Analysis
Source: Front Aging Neurosci. 2022 Jun 30;14:939848. doi: 10.3389/fnagi.2022.939848 (PMC9284274; doi:10.3389/fnagi.2022.939848)
Supplement: Supplementary Material — Search strategy. [file Data_Sheet_1.ZIP › Supplementary Material/Supplementary Table 2.docx]

**Supplementary Table 2** Meta-Regressions for the incidence of PNDs

| logrr | exp(b) | Std. Err. | t | P>\|t\| | [95% Conf. Interval] | |
| --- | --- | --- | --- | --- | --- | --- |
| dose | .3467454 | .1949559 | -1.88 | 0.118 | .0817196 | 1.471279 |
| surgery | .511698 | .3465075 | -0.99 | 0.368 | .0897483 | 2.917436 |
| age | .7395386 | .226275 | -0.99 | 0.369 | .3368073 | 1.623828 |
| _cons | 2.016479 | 1.390129 | 1.02 | 0.356 | .3427458 | 11.86357 |
