# Supplementary figures and images for: Effects of Glucocorticoids on Postoperative Neurocognitive Disorders in Adult Patients: A Systematic Review and Meta-Analysis
Source: Front Aging Neurosci. 2022 Jun 30;14:939848. doi: 10.3389/fnagi.2022.939848 (PMC9284274; doi:10.3389/fnagi.2022.939848)

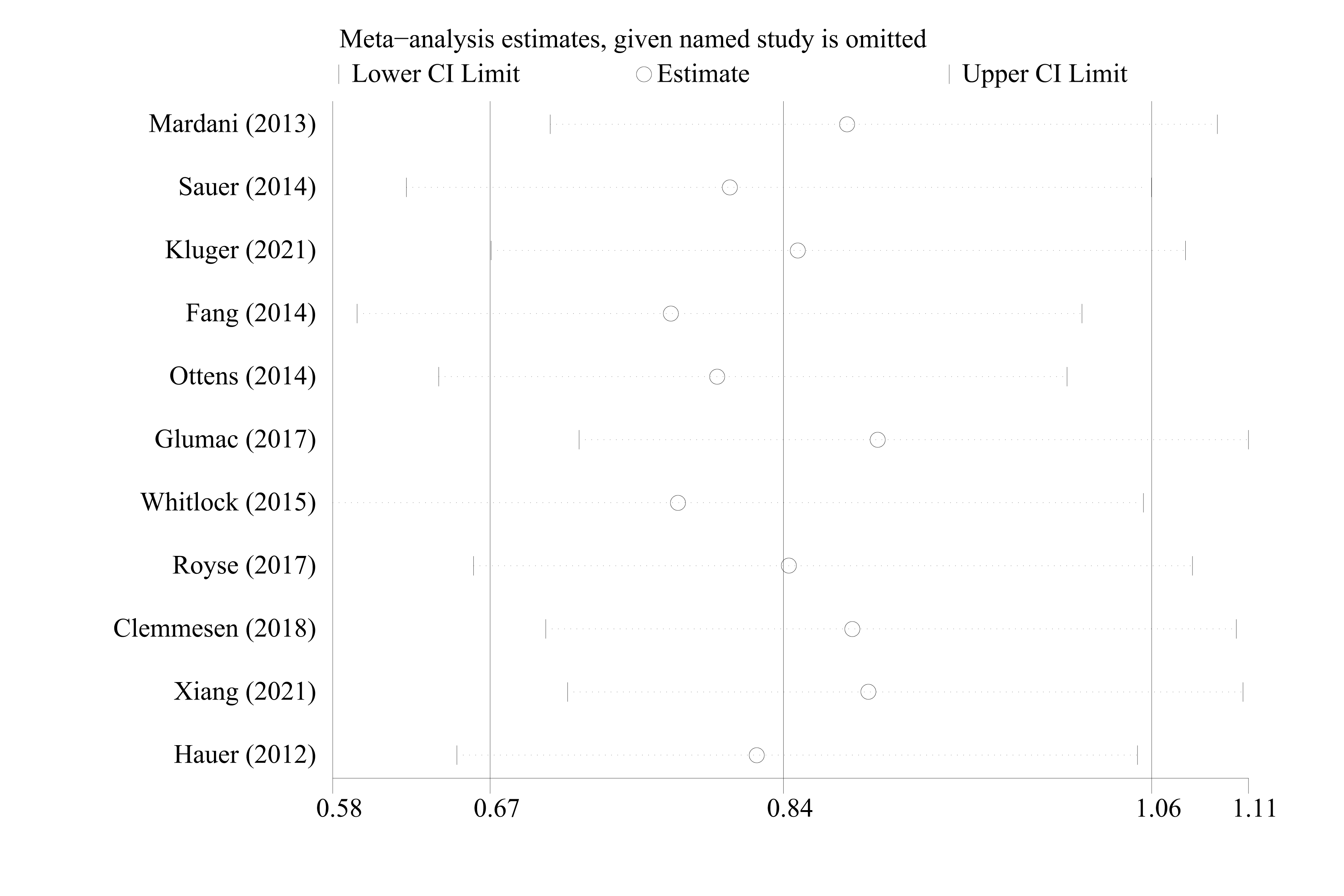

Supplement: Supplementary Material — Search strategy. [file Data_Sheet_1.ZIP › Supplementary Material/Supplementary Figure 1.tif]

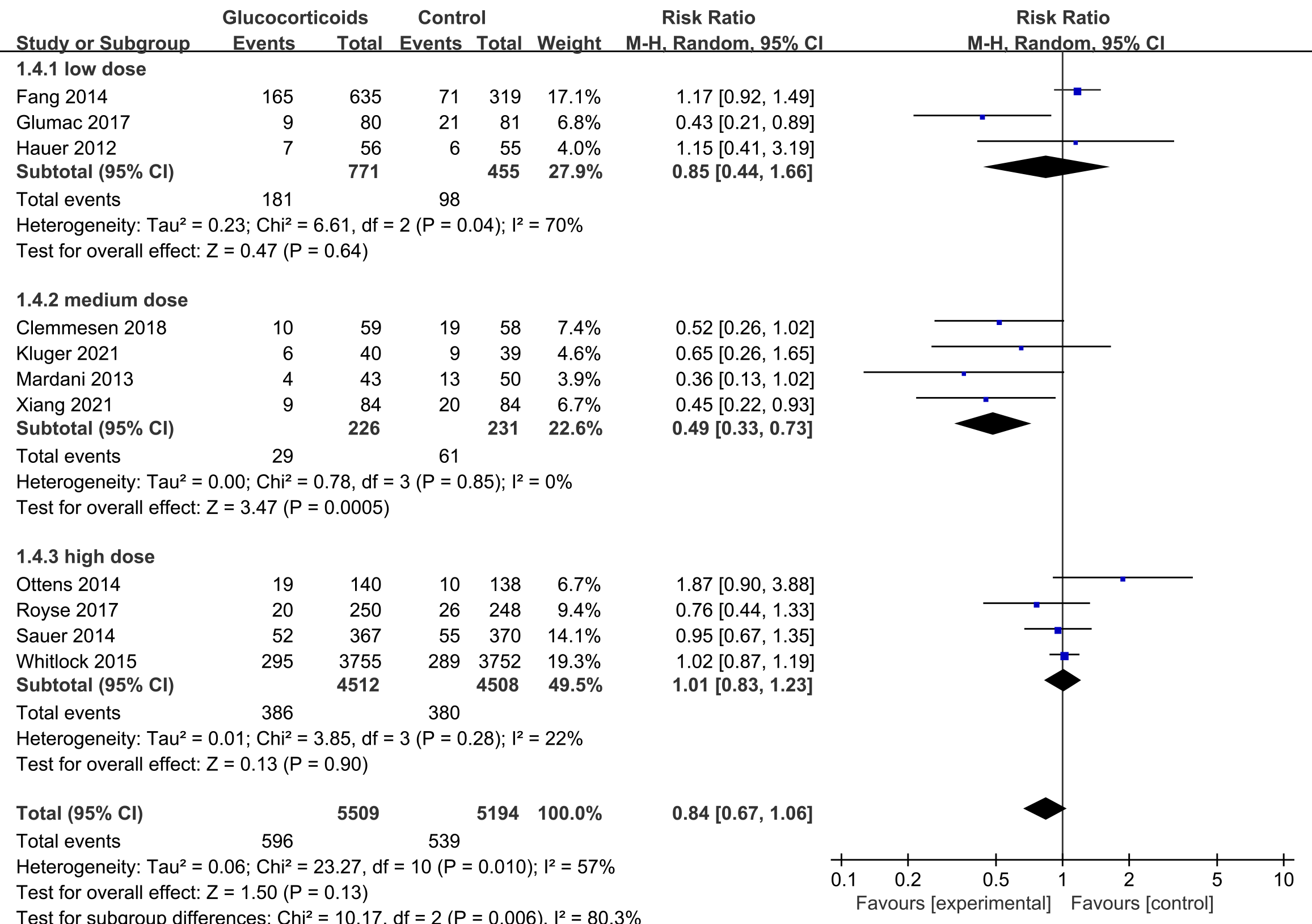

Supplement: Supplementary Material — Search strategy. [file Data_Sheet_1.ZIP › Supplementary Material/Supplementary Figure 2.tif]

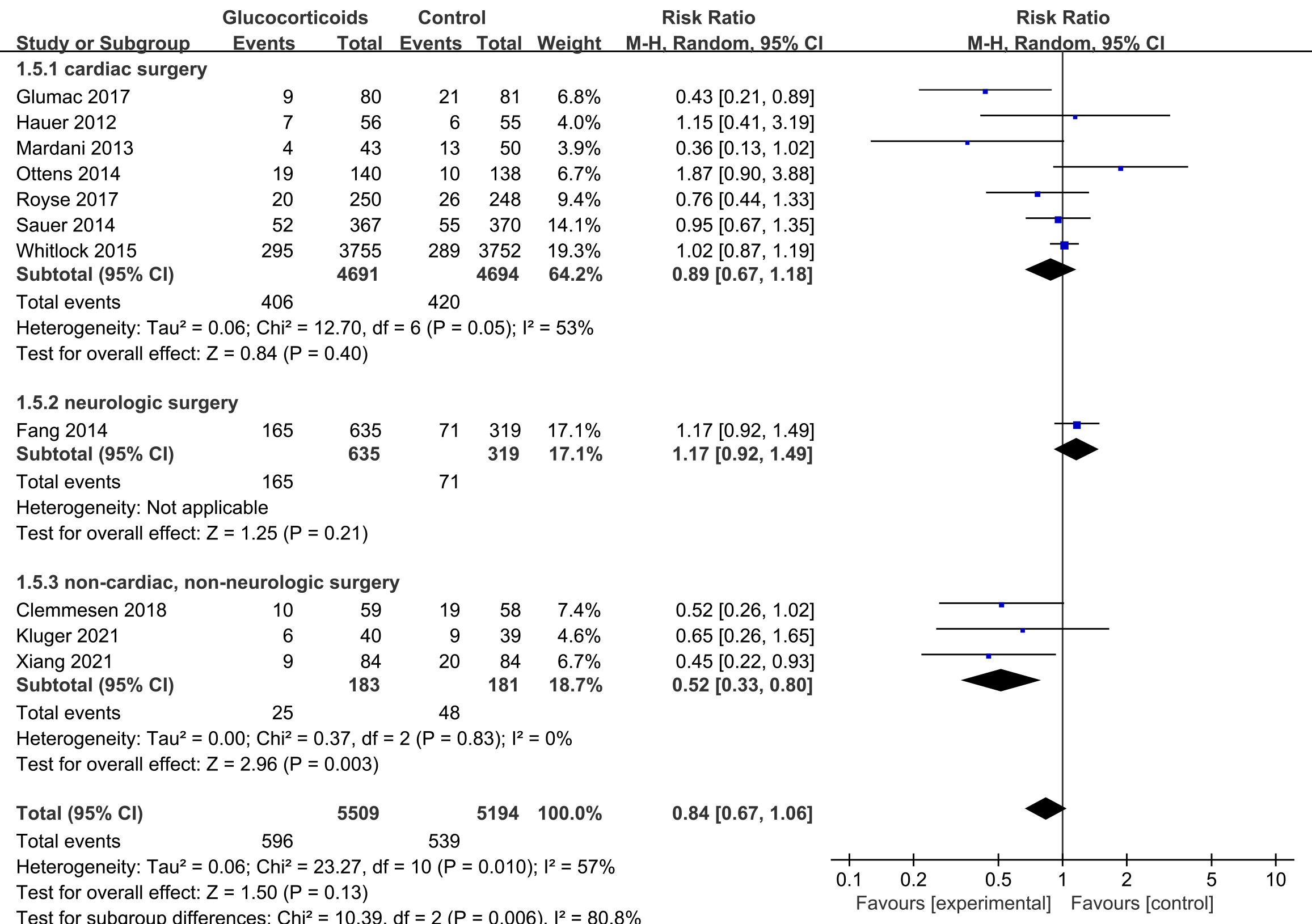

Supplement: Supplementary Material — Search strategy. [file Data_Sheet_1.ZIP › Supplementary Material/Supplementary Figure 3.tif]

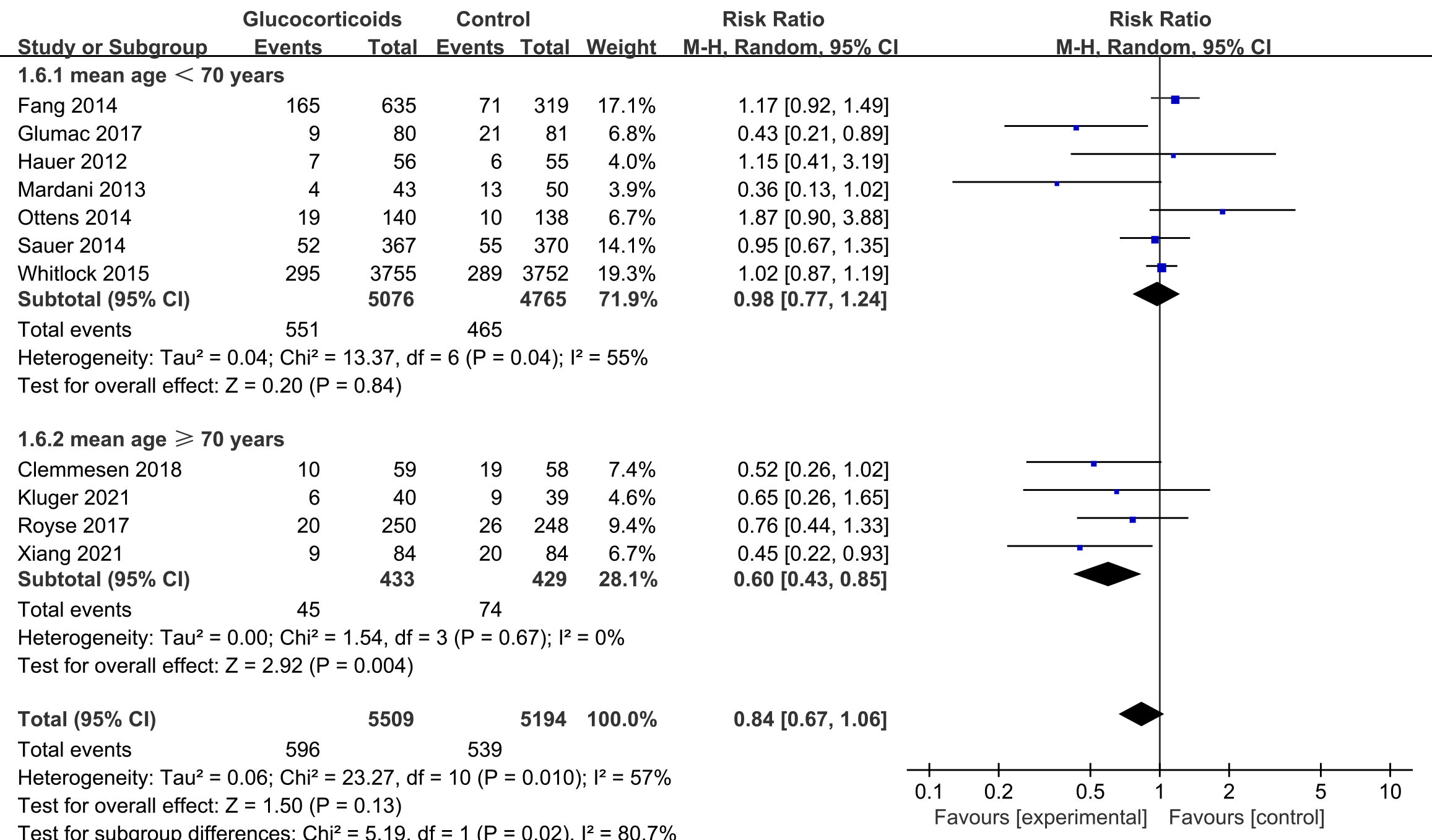

Supplement: Supplementary Material — Search strategy. [file Data_Sheet_1.ZIP › Supplementary Material/Supplementary Figure 4.tif]
